# Molecular, Microbial, and Ecological Drivers of Duckweed Phytoremediation in Aquatic Environments

**DOI:** 10.3390/biology15120963

**Published:** 2026-06-19

**Authors:** Doni Thingujam, Antonino Malacrinò, Karolina M. Pajerowska-Mukhtar, M. Shahid Mukhtar

**Affiliations:** 1 Department of Biological Sciences, Clemson University, Clemson, SC 29634, USA; dthingu@clemson.edu (D.T.); amalacr@clemson.edu (A.M.); kmukhta@clemson.edu (K.M.P.-M.); 2 Department of Genetics & Biochemistry, Clemson University, Clemson, SC 29634, USA

**Keywords:** bioeconomy, microbiome, detoxification, heavy metals, wastewater, ecosystems, biomass

## Abstract

Aquatic ecosystems are under severe stress from a diverse combination of contaminants, including heavy metals, pesticides, pharmaceuticals, and microplastics, driven by rapid industrialization, intensive agriculture, and urbanization. Globally, 80% of wastewater remains untreated, and conventional systems often fail to address emerging contaminants. Consequently, toxic heavy metals like lead and mercury can persist in water sources for decades. In response, phytoremediation has emerged as a scalable, eco-friendly, nature-based alternative. Among phytoremediation agents, duckweeds are increasingly recognized for their rapid growth, simple morphology, and continuous water-column contact. This review outlines the landscape of duckweed-based remediation, detailing molecular detoxification pathways and the synergistic role of associated microbiomes in enhancing environmental cleanup. Evidence indicates that contaminant removal is often supported by plant-microbe interactions. Despite extensive laboratory validation, field-scale implementation remains constrained by environmental complexity, pollutant mixtures, and variable climatic conditions. Furthermore, while duckweed systems hold promise within circular bioeconomy frameworks, converting wastewater into nutrient-rich biomass, contaminant accumulation in plant tissues raises concerns about biomass utilization and contaminant carryover. Addressing these challenges requires an integrative approach that links molecular detoxification, ecological interactions, and engineered system design to realize the full potential of duckweeds for sustainable aquatic pollution management.

## Simple Summary

Aquatic pollution from diverse contaminant sources has outpaced the capacity of conventional treatment systems, creating urgent demand for nature-based remediation alternatives. Duckweeds (Lemnaceae) represent a compelling option, combining rapid growth and broad pollutant uptake with the ability to support functionally specialized microbial communities that enhance contaminant transformation. Duckweeds are not only passive sinks for pollutants; they exhibit dynamic molecular and physiological adaptations to chemical stress that vary meaningfully across species, genotypes, and environmental contexts. Field-scale application, however, remains constrained by pollutant mixture complexity, climatic variability, and system design factors that laboratory assays consistently underestimate. The potential to integrate remediation with circular bioeconomy outputs, including animal feed, biofuel, and biofertilizer, is promising but contingent on resolving contaminant carryover risks in harvested biomass. Understanding the full potential of duckweed systems will require integrative, multi-scale research frameworks that connect molecular detoxification, ecological interactions, and engineered system design.

## 1. Introduction

Aquatic bodies are getting polluted at a greater pace than at any other point in recorded history [1]. This is driven by rapid industrialization, intensive agricultural practices, and expanding urbanization. According to UN-Water, approximately 80% of global wastewater is discharged untreated into the environment, resulting in widespread degradation of aquatic ecosystems, threats to human health, and accelerating loss of biodiversity [1]. These contaminants span a broad spectrum from heavy metals and synthetic pesticides to pharmaceuticals, nutrients, microplastics, and per- and polyfluoroalkyl substances (PFAS), many of which resist conventional removal and accumulate progressively in food chains [2,3,4,5]. Heavy metals such as lead, cadmium, chromium, and mercury can remain in surface and groundwater for decades, also posing serious risks as traditional treatment systems fail to effectively remove them from industrial effluents, sewage, and farm runoff [2,3,4,5]. Addressing this accelerating crisis demands scalable, effective, and environmentally sound remediation strategies. Conventional approaches to wastewater treatment encompass a range of physical, chemical, and biological methods. Techniques such as electrocoagulation, membrane filtration, chemical precipitation, and advanced oxidation processes have been widely deployed. However, these methods carry significant limitations, including high operational costs, secondary pollutant generation, and energy-intensive requirements that restrict their adoption in low-income regions [6]. Traditional treatment systems are generally not well-equipped to efficiently remove emerging contaminants from complex wastewater matrices [7,8].

Phytoremediation uses living plants and their associated root-zone microbiota to remove, immobilize, or detoxify pollutants and has emerged as a particularly compelling alternative to conventional approaches. Because of its eco-friendly nature, phytoremediation holds a clear advantage over traditional physicochemical techniques, which often produce harmful by-products detrimental to both biological systems and the broader environment [9]. The approach encompasses several complementary mechanisms, including phytoextraction, phytostabilization, rhizofiltration, phytodegradation, and phytovolatilization, enabling plants to intercept contaminants across diverse environmental matrices. Phytoremediation has gained recognition as a low-impact, nature-based solution effective against a wide range of aquatic and terrestrial pollutants, including pharmaceuticals and heavy metals [10]. Crucially, unlike energy-intensive engineering solutions, phytoremediation can be implemented at scale with minimal infrastructure, offering considerable socioeconomic advantages for communities in the developing world [11,12,13].

**Figure 1:**
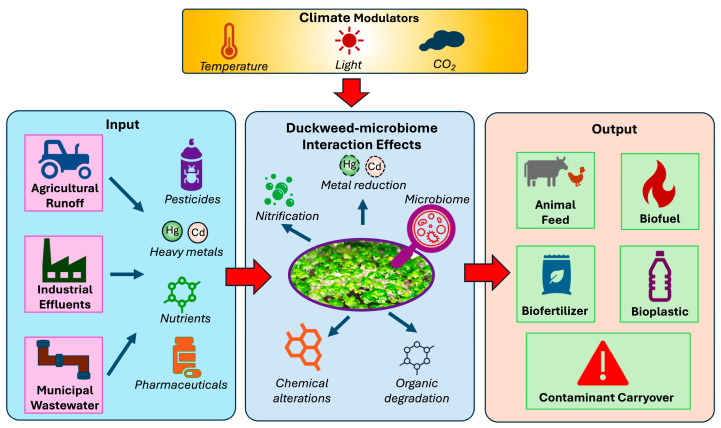
Conceptual overview of duckweed-based phytoremediation systems and associated outputs. Agricultural runoff, industrial effluents, and municipal wastewater introduce contaminants, including pesticides, heavy metals, nutrients, and pharmaceuticals, into aquatic environments. Duckweed-microbiome interactions mediate contaminant removal through processes such as nitrification, metal reduction, chemical transformation, and organic degradation, while environmental modulators, including temperature, light, and CO_2_, influence remediation efficiency. Treated biomass can be utilized for animal feed, biofuel, biofertilizer, and bioplastic production. However, contaminant carryover within harvested biomass remains a major challenge for downstream applications. The bioeconomy outputs depicted animal feed, biofuel, biofertilizer, and bioplastic are conditional endpoints, feasible only where contaminant burden in harvested biomass has been assessed and managed. Biomass harvested from high-load wastewater streams containing heavy metals, antibiotics, or persistent xenobiotics may require pretreatment, contaminant removal steps, or controlled disposal rather than immediate reuse.

Among the most promising candidates for aquatic phytoremediation are the duckweeds (family Lemnaceae), a group of small, free-floating angiosperms distributed across freshwater systems worldwide. Duckweeds possess one of the fastest growth rates among all flowering plants and can accumulate large quantities of biomass over short periods, while also demonstrating exceptionally high adaptability to a wide range of environmental conditions [14,15]. Moreover, their phytoremediation efficacy is broad-spectrum. For example, species of *Lemna* have demonstrated the capacity to assimilate nitrogen and phosphorus, sequester heavy metals, and intercept emerging contaminants such as pharmaceuticals and pesticides [16]. Beyond remediation, duckweed biomass generated during pollutant uptake can serve as feedstock for biofuels, animal feed, and other value-added applications, positioning these plants as contributors to a circular bioeconomy [15]. Despite these advantages, translating laboratory-scale successes into reliable field applications requires a deeper, multi-scale understanding of the molecular, physiological, and ecological mechanisms that govern duckweed-pollutant interactions. While existing literature has advanced our understanding of nutrient dynamics and heavy metal uptake [15] and has explored duckweed species diversity in the context of contaminant biomonitoring [17], these contributions have largely addressed individual dimensions of remediation in isolation. A synthesis that connects sub-cellular detoxification pathways, microbiome-mediated processes, and the safe valorization of pollutant-laden biomass within a coherent conceptual framework remains lacking. In this review, we synthesize the current knowledge on duckweed-based remediation, focusing on molecular detoxification mechanisms, species diversity, interactions with the microbiome, and the challenges of translating laboratory findings to field applications (Figure 1). Overall, our review highlights that duckweed remediation can operate through multi-scale biological processes that link molecular detoxification, ecological interactions, and engineered system design. We emphasize the need for integrative approaches to maximize the application of duckweed-based systems for environmentally sustainable remediation of aquatic pollutants.

## 2. Duckweeds as Dynamic Interfaces in Aquatic Pollution Management

Aquatic ecosystems are increasingly challenged by complex mixtures of nutrients, heavy metals, pharmaceuticals, antibiotics, and industrial chemicals that persist at low but biologically active concentrations [18,19]. In comparison to historical point-source pollution, modern contamination is diffuse, chronic, and sustained by agricultural runoff, wastewater reuse, and urban effluents [19,20,21]. Conventional physicochemical wastewater treatment methods remain effective for bulk nutrient removal, although they are costly, energy-intensive, and poorly suited for low-concentration micropollutants such as antibiotics, cytostatics, and endocrine disruptors [22,23,24,25]. This limitation raises a fundamental question: what biological systems can operate effectively at the interface of chemical complexity, low-dose exposure, and ecological variability? Duckweeds occupy a unique ecological niche at the water-air interface, where dissolved pollutants are intercepted before being transported downstream. Historically, duckweeds have been extensively explored as phytoremediation agents due to their rapid clonal growth, continuous exposure of photosynthetic tissue to the water column, and high surface-area-to-volume ratio, which enable fast uptake and transformation of contaminants [14,26]. However, recent experimental and pilot-scale studies increasingly challenge the notion that duckweeds function only as passive pollutant sinks. Chronic exposure to pharmaceuticals such as oxytetracycline, tetracyclines, and metformin induces coordinated changes in growth, nutrient assimilation, and physiological state instead of uniform toxicity, indicating active stress integration and regulation [23,24,27]. Moreover, duckweed-mediated remediation can reduce ecological harm to non-target organisms exposed to pharmaceutical contaminants, suggesting system-level buffering rather than simple accumulation [28].

A practical constraint of surface-floating duckweeds is that direct contaminant contact is limited to the uppermost water layer. Uptake from deeper water layers depends on passive diffusion, convective transport toward the surface, and the physical extent of the root system. The roots of *Lemna* and *Spirodela* species can extend several centimeters below the frond mat, a distance that substantially increases the effective remediation zone and allows interception of dissolved metals and organics before they partition into deeper sediments [29]. Externally, root-surface iron plaques serve as a first-line trapping mechanism for arsenic and lead [29], and chelating agents that modify plaque formation can further regulate metal bioavailability and uptake efficiency [29,30]. In engineered systems, these passive limitations are substantially overcome through hydraulic design. In baffled reactors, flow-through ponds, and staged treatment wetlands, controlled water circulation and optimized hydraulic residence time actively deliver contaminants from deeper layers to the duckweed rhizosphere [26,31]. Van Dyck et al. (2021) [26] demonstrated that hydrodynamic parameters, including flow rate and residence time, are among the strongest determinants of *Lemna minor* growth and pollutant contact, emphasizing that system engineering rather than plant biology alone defines the effective remediation depth in field-scale applications. Pilot-scale evidence further confirms that such engineered configurations can achieve simultaneous nutrient removal, carbon capture, and biomass production even under variable contaminant loads [31].

Heavy metal remediation studies reinforce this interpretation. Duckweed-mediated uptake of lead, cadmium, chromium, arsenic, and other metals is strongly influenced by environmental context, including pH, contaminant concentration, and species composition [32,33]. Polyculture systems, for instance, can enhance Pb removal compared with single-species setups, indicating emergent functional properties that cannot be predicted from individual species alone [33]. Chemical modifications such as oxalic acid treatment further enhance metal phytoextraction efficiency, consistent with the broader principle that effective plant-based remediation depends on regulated physiological responses rather than passive sorption alone [30]. Importantly, duckweed systems have shown potential to operate at scales beyond laboratory assays. Simultaneous nutrient removal, carbon capture, and biomass production have been reported through pilot-scale duckweed-CO_2_ wastewater treatment platforms, demonstrating metabolic integration across system components rather than single-function remediation [31]. In agricultural contexts, floating duckweed reduces ammonia volatilization while improving nitrogen use efficiency, suggesting that duckweeds influence biogeochemical fluxes well beyond contaminant uptake [34]. Likewise, biomass accumulation and nitrogen content in *Wolffia* species vary predictably with wastewater strength, indicating physiological tuning to nutrient regimes [35]. These findings collectively raise an intriguing question: are duckweeds best understood as experimental phytoremediation tools, or as systems capable of coordinating physiological responses to chemical stress? Their documented ability to remediate pollutants while maintaining growth and system stability suggests that duckweeds can manage physiological responses to environmental stress [27,28,36].

## 3. Species Diversity and Phenotypic Plasticity as Functional Assets

### 3.1. Species- and Genotype-Dependent Remediation Performance

Different duckweed species have been known to remediate a wide range of pollutants, including heavy metals, pharmaceuticals, pesticides and herbicides, emerging organic contaminants, industrial chemicals and surfactants, dyes, cyanotoxins, microplastics, nanomaterials, and contaminants of emerging concern (CECs) through various mechanisms. These include rhizofiltration (adsorption and absorption of contaminants from water into roots or fronds), bioaccumulation (uptake and storage inside plant tissues), biodegradation (breakdown of organic pollutants by duckweed-associated enzymes or microbes), and photodegradation enhancement (where duckweed modifies light exposure or pH to facilitate chemical breakdown). Additionally, duckweed can promote the precipitation or complexation of metals, alter the speciation of contaminants, and support rhizosphere microbial communities that further degrade pollutants. These mechanisms operate in parallel or sequentially, depending on the contaminant type and environmental conditions. Comprehensive species and strain comparisons across a wide range of contaminant classes with mechanisms are provided in Table 1.

Among the duckweed species, *L. minor* is a primary model in both laboratory research and applied phytoremediation. However, several studies across metals, nutrients, dyes, antibiotics, and emerging contaminants consistently show that remediation performance varies strongly across species of the genera *Lemna*, *Spirodela*, *Landoltia*, and *Wolffia*, depending on contaminant chemistry and environmental context [36,59,68,71,79,109]. These differences often reflect trade-offs between growth resilience and contaminant accumulation. *Spirodela polyrhiza* often sustains higher biomass under metal stress, whereas *L. minor* frequently exhibits higher metal accumulation per unit biomass, particularly for cadmium and nickel, reflecting trade-offs between stress tolerance and extraction efficiency [56,68,71]. Such trade-offs imply that accumulation efficiency at the tissue level may come at the cost of long-term growth stability under chronic exposure. Similar species-specific patterns have been reported for cobalt and nickel in *Landoltia punctata*, where removal efficiency is tightly coupled to photosynthetic performance and antioxidant capacity [54]. For *L. punctata*: (i) Guo et al. (2017) [54] tested cobalt and nickel phytoremediation using hydroponic batch experiments; removal efficiency was 58.63% and 56.23%, respectively. The photosynthesis rate, chlorophyll content, and Rubisco activity were greatly impacted by high concentrations of these metals. (ii) Xu et al. (2018) [46] performed comparative transcriptomics after cadmium exposure at 20 μM over 7 days, revealing coordinated upregulation of iron-regulated transporters (IRT), metal transport proteins (MTP), multidrug resistance-associated proteins (MRP), and yellow stripe-like transporters (YSL) consistent with enhanced vacuolar sequestration of cadmium. In contrast, proteins associated with cadmium efflux to the apoplast, including pleiotropic drug resistance 8 (PDR8), plant cadmium resistance 2 (PCR2), and heavy metal ATPase 2/4 (HMA2/4), exhibited little or no transcriptional response, indicating intracellular compartmentalization. (iii) Wang et al. (2022) [45] conducted a large-scale screening of cadmium accumulation across four *L. punctata* accessions, reporting bioconcentration factors ranging from 2511.1 to 30,641.01 mg/kg with an increase in Cd treatment levels from 0.3 to 20 mg/L, with ultrastructural deformation of chloroplasts at 2 mg/L. (iv) Wang et al. (2021) [53] used sequential extraction to characterize cadmium chemical forms, finding that 21–54% of accumulated Cd resided in cell wall-bound fractions, with smaller vacuolar and cytoplasmic pools. (v) Wang et al. (2024) [44] showed that ammonium-dominated nitrogen speciation enhanced cell wall immobilization of cadmium relative to nitrate conditions, providing a mechanistic link between nitrogen form and metal detoxification efficiency. In contrast, *L. gibba* shows strong but pollutant-specific tolerance, including efficient accumulation of boron, chromium, cadmium, and synthetic dyes, with measurable ultrastructural stress responses [59,67,102,112]. Size is not always predictive of phytoremediation efficacy; in some cases, small species like *Wolffia arrhiza* demonstrate superior removal of emerging contaminants such as bisphenol A, diethylstilbestrol, estrone, estradiol, etc., compared to larger duckweed taxa. [109]. Notably, even closely related species can diverge in their capacity to remove contaminants of emerging concern. Comparative experiments demonstrate that *W. arrhiza* and *L. minor* differ markedly in removal efficiency depending on pollutant concentration and matrix composition, challenging assumptions that smaller body size necessarily limits remediation potential [109]. Moreover, mixed-species cultures can outperform monocultures for specific pollutants. For instance, mercury bioaccumulation is enhanced in *L. minor*-*S. polyrhiza* co-cultures relative to single-species systems, suggesting functional complementarity rather than redundancy [56]. These findings raise a central question: why do most remediation systems continue to rely on single-species designs for chemically complex waste streams? The practice of treating duckweeds as interchangeable species neglects their functional diversity, thereby limiting the development of precisely fitted phytoremediation systems.

Beyond interspecific differences, substantial intraspecific variation in remediation capacity has been documented within individual duckweed species, a dimension frequently overlooked in single-genotype remediation studies. Rzodkiewicz and Turcotte (2024) [113] demonstrated genotype-dependent responses to microcystin-LR across *Spirodela* lineages, showing that genetic background within a species can shift pollutant tolerance thresholds as significantly as species identity itself. Similarly, Chávez et al. (2026) [114] documented heritable and repeatable transgenerational fitness variation in *S. polyrhiza* genotypes under copper stress, wherein offspring of copper-stressed ancestors displayed enhanced resistance to recurring copper exposure, potentially through improved photosystem II protection, although this adaptive benefit was frequently accompanied by reduced fitness under non-stress conditions. The remediation capacity of *L. minor* has been documented across a remarkable range of contaminants, including heavy metals [29,39,50,51,57,58,61,62,63,71,72,74,76], pharmaceuticals [23,27,28,78,81,83,84,87], dyes [103,104], micropollutants [92,97,98,107], and mycotoxins [91], and its species-specific uptake efficiency relative to other macrophytes, such as Eichhornia crassipes, has been established for several metal and organic contaminants [57,62]. This intraspecific functional diversity has direct implications for phytoremediation design: screening multiple genotypes within a single species may reveal high-performing lineages with substantially enhanced accumulation or tolerance capacity.

### 3.2. Passive Uptake and Active Reprogramming in Duckweed Epigenetic Plasticity

Beyond species identity, duckweed remediation capacity is regulated by epigenetic mechanisms and phenotypic plasticity. Transcriptomic analyses reveal that pollutant exposure triggers coordinated regulation of transporter families, antioxidant enzymes, nitrogen assimilation pathways, and detoxification systems, indicating active physiological reprogramming rather than passive uptake [46,115]. In *L. punctata*, cadmium exposure induces the expression of genes associated with hyperaccumulation, redox control, and carbon allocation, linking stress tolerance to broader metabolic remodeling [46,54]. Metal-binding proteins further contribute to species-specific responses. Expansion and diversification of metallothionein gene families in *S. polyrhiza* suggest specialized roles in metal chelation and sequestration [116]. Functional characterization of SpMT2a further demonstrated its active involvement in cadmium and copper tolerance through heterologous expression assays, supporting a direct role for metallothioneins in heavy metal detoxification and stress adaptation [116]. Importantly, tolerance is not uniform even within species. Genotype-dependent responses to microcystin-LR, a toxic cyanobacterial hepatotoxin commonly released during harmful algal blooms in eutrophic aquatic systems exposure have been documented across *Spirodela* lineages, demonstrating that genetic background alone can shift toxicity thresholds [113]. Emerging evidence further suggests that duckweed stress responses can persist across generations. *L. minor* exhibits enhanced tolerance to cyanobacterial exudates, which may contain bioactive or toxic metabolites released by cyanobacteria, across generations, suggesting that stress memory and heritable regulatory changes might contribute to long-term acclimation through transgenerational plasticity [117]. Such findings challenge the assumption that species-level identity alone predicts remediation performance.

In the context of duckweed phytoremediation, epigenetic plasticity refers to heritable changes in gene expression patterns that occur without alteration of the underlying DNA sequence. The most thoroughly characterized epigenetic mechanism in duckweeds is DNA methylation, the covalent addition of methyl groups to cytosine residues, predominantly in CG, CHG, and CHH sequence contexts (where H = A, T, or C) [118]. Van Antro et al. (2023) [118] demonstrated in *L. minor* that environmentally induced methylation changes, particularly in CG and CHG contexts, persist for 3–12 clonal generations following removal of the initial stress trigger. These heritable methylation signatures are frequently enriched in transposable element (TE) regions, consistent with the general principle that TE methylation is a major vehicle for stable epigenetic inheritance across cell divisions and asexual reproduction cycles. In plants, stress-induced DNA methylation responses vary substantially across species and environmental conditions. While Arabidopsis typically shows stress-responsive changes predominantly in the CHH context, duckweeds such as *L. minor* display a distinct pattern in which CG and CHG contexts are the primary responsive methylation marks [118]. This contrasts with the reduced and altered methylation landscape observed in *S. polyrhiza*, where partial loss of key DNA methylation pathway components has led to genome-wide hypomethylation [118]. In *L. minor*, however, relatively high and conserved methylation levels across all contexts, together with the presence of intact homologs of core methylation machinery genes, indicate a functional and plant-typical epigenetic system [118]. Evidence further suggests that while CHH methylation may act as a rapid and transient stress-responsive mark, prolonged or multigenerational exposure can shift epigenetic responses toward more stable CG- and CHG-associated changes. Similar patterns have been reported in long-term stress experiments in Arabidopsis, where multigenerational exposure resulted primarily in CG and CHG differentially methylated regions with minimal CHH involvement [118]. This leads to a broader, unresolved question: are epigenetic mechanisms enabling rapid acclimation being overlooked in duckweed-based remediation systems? The clonal reproduction of duckweeds provides a distinctive framework to separate genetic, regulatory, and environmental drivers of stress tolerance. Nevertheless, key mechanistic validations, such as functional analyses of detoxification genes and comparative genotype studies, along with explorations of stress memory, are still largely absent. Until systematic genotype screening, functional validation of detoxification genes, and tests of stress memory are incorporated, duckweed diversity will remain an unexplored asset for next-generation phytoremediation design.

## 4. Core Molecular Mechanisms of Uptake and Detoxification

Duckweed responses to contaminants operate through two interconnected but analytically distinct categories of mechanisms. The first encompasses direct phytoremediation mechanisms that physically remove, sequester, or chemically transform contaminants, thereby reducing their bioavailability in the water column. These include transporter-mediated uptake, vacuolar sequestration, cell wall immobilization via pectin thickening and iron plaques, chelation by phytochelatins and metallothioneins, and enzymatic biotransformation of organic pollutants through the Phase I–III green liver pathway (Table 2). The second category comprises stress tolerance mechanisms, physiological responses that protect the plant from contaminant-induced damage and maintain growth and metabolic function under exposure, including ROS scavenging, antioxidant enzyme induction, and epigenetic regulatory responses (Table 2). While both categories are necessary for sustained remediation performance, tolerance mechanisms enable plant survival but do not themselves remove contaminants from water; only the first category achieves actual pollutant load reduction.

### 4.1. Transport, Sequestration, and Immobilization

**Figure 2:**
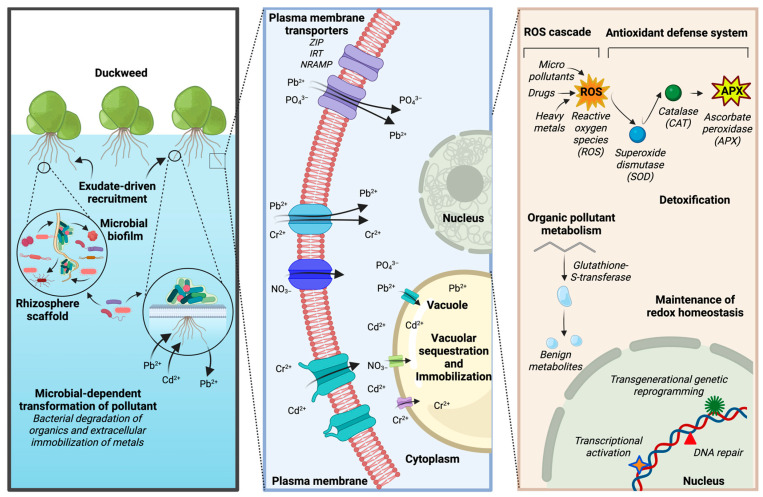
Molecular and cellular mechanisms underlying duckweed phytoremediation. (**Left**) Rhizosphere scaffold showing exudate-driven microbial biofilm recruitment and microbial-dependent pollutant transformation, including bacterial degradation of organic pollutants and extracellular immobilization of metal ions. (**Center**) Plasma membrane transporter families (ZIP/IRT importers, NRAMP permeases) mediate competitive metal ion entry alongside nutrient channels; internalized metals are routed to vacuolar sequestration or cytoplasmic chelation by phytochelatins and metallothioneins. (**Right**) Contaminant exposure triggers ROS production, countered by a coordinated antioxidant enzyme network (SOD, CAT, APX); organic pollutants undergo glutathione-S-transferase-mediated biotransformation toward benign metabolites; sustained stress induces transcriptional activation and epigenetic changes in the nucleus, sustaining heritable transgenerational stress memory.

The entry of pollutants into duckweed tissues is a highly regulated process involving a coordinated network of membrane transporters. Studies show that heavy metal ions frequently compete with essential nutrients for entry through shared, high-affinity membrane transporters [40,63]. Duckweed-mediated phytoremediation is initiated by rapid pollutant uptake, yet a central question remains: how do these plants absorb high pollutant loads while maintaining high growth rates (Figure 1)? Evidence suggests that different plants, including duckweed, utilize existing nutrient transport systems for contaminant entry that frequently overlap with nutrient acquisition pathways. Metal ions such as cadmium, lead, chromium, cobalt, and nickel enter duckweed tissues via divalent cation transporters and nutrient-associated channels, as demonstrated across *Lemna*, *Spirodela*, and *Landoltia* species [40,41,45,50,63]. Three major transporter families mediate metal ion entry into duckweed tissues (Figure 2). ZIP (Zinc/Iron Permease) and IRT (Iron-Regulated Transporter) family proteins are high-affinity divalent cation importers responsible for cadmium, zinc, and manganese uptake; their substrate promiscuity allows toxic metals to enter via pathways evolved for essential micronutrient acquisition [40,63]. NRAMP (Natural Resistance Associated Macrophage Protein) family permeases facilitate broad-specificity metal transport across the tonoplast and plasma membrane and have been implicated in cadmium, manganese, and iron homeostasis in related monocots [63]. HMA-type (Heavy Metal ATPase) P-type ATPases are responsible for loading metals into the vacuole and have been partially annotated in the *S. polyrhiza* genome [119]; their upregulation under cadmium and nickel stress in *L. punctata* [46] and *L. minor* [68] links transporter expression directly to the sequestration strategy. The ratio of nitrate to ammonium in the growth medium modulates the rate of cadmium entry through these channels [44,115], with ammonium-dominated conditions enhancing cell wall immobilization relative to vacuolar uptake [44], demonstrating that nutrient form directly controls the partitioning of contaminant fate within the plant. The dynamics of cadmium uptake are intricately coordinated with the expression of major nitrogen assimilation genes, suggesting that nutrient availability, specifically the ratio of nitrate to ammonium, directly dictates the rate of pollutant entry [44,115]. Once internalized, duckweed detoxifies metals through a sequestration strategy to protect its metabolic core (Figure 2). This includes vacuolar sequestration, where ions are pumped into specialized compartments and transitioned into less reactive stored states [45,46].

Metals are detoxified through molecular chelation by thiol-rich peptides and proteins, notably phytochelatins and metallothioneins, whose gene families are expanded and transcriptionally responsive in duckweed genomes [46,58,116]. Externally, duckweed utilizes pectinous cell wall thickenings and root-surface iron plaques to trap metals like lead and arsenic [29,70,120]. Phytochelatin (PC) synthesis is catalyzed by phytochelatin synthase (PCS), a gamma-glutamylcysteine dipeptidyl transpeptidase that uses glutathione (GSH) as the substrate for iterative peptide extension, generating PC2 through PC6 depending on the metal load and GSH availability. Török et al. (2015) [58] demonstrated in aquatic plants, including duckweed, that phytoremediation capacity is positively correlated with the degree of PC polymerization. Higher chain length PCs (PC4-PC6) bind metals with greater affinity and are more effective at sequestration into vacuolar compartments. This finding has direct implications for system design: cultivation conditions that support GSH biosynthesis (e.g., adequate sulfur supply) may enhance phytoremediation performance by enabling higher-degree PC synthesis. Metallothionein SpMT2a, characterized by Pakdee et al. (2022) [116] in *S. polyrhiza*, binds cadmium and copper through cysteine residues in its metal-binding domains and confers tolerance when expressed heterologously in yeast, establishing direct functional evidence for its role in heavy metal detoxification, not merely correlational expression data. However, it is unclear to what extent these chelation systems are constitutively active or inducible, and how their efficacy scales under variable pollutant conditions. Vacuolar sequestration and cell wall immobilization further buffer cytosolic toxicity [17,53], but the energetic and regulatory trade-offs of maintaining these storage sinks under chronic exposure remain unresolved. These questions are particularly relevant for long-term remediation systems where pollutant influx is continuous rather than periodic. A significant gap remains regarding the specificity and competition between different toxic ions and essential micronutrients at the level of the transporter. Predictive modeling of phytoremediation in mixed-waste settings is currently limited by the absence of high-resolution kinetic models that can account for interference from complex, multi-metal effluents on specific cellular uptake pathways.

### 4.2. Redox Homeostasis and Genomic Stability

Both inorganic metals and organic xenobiotics can induce oxidative stress in duckweeds, similar to many plant species, positioning redox homeostasis as a central integrator of detoxification responses. Exposure to pollutants such as metals, antibiotics, and herbicides invariably triggers the production of Reactive Oxygen Species (ROS), which can damage proteins and DNA [80,121]. Duckweed integrates its detoxification with a robust antioxidant system, utilizing superoxide dismutase, catalase, ascorbate peroxidase, and glutathione-dependent enzymes to maintain redox balance [54,73] (Figure 2). Transcriptomic analyses further reveal coordinated induction of redox-related genes alongside transporters and chelation pathways, highlighting tight regulatory coupling between uptake and detoxification [46]. However, is oxidative signaling simply defensive, or does it actively instruct detoxification capacity and tolerance thresholds? The extent to which ROS function as second messengers shaping transcriptional reprogramming remains poorly defined, particularly in non-model aquatic plants. Redox signaling intersects with growth regulation, photosynthesis, and nutrient metabolism, shaping tolerance thresholds and recovery capacity [44,54]. This response is further modulated by signaling molecules like melatonin, which has been shown to enhance growth and metabolites under stress by recalibrating the plant’s antioxidant defense [122]. The ability of *L. minor* to maintain genetic stability, preserving DNA integrity even when exposed to binary mixtures of cadmium and nickel, highlights an evolutionary resilience that distinguishes it from more sensitive aquatic species [68]. Genetic stability refers here specifically to the maintenance of DNA template integrity, the degree to which the DNA sequence remains undamaged and replicable under genotoxic stress, rather than to chromosomal structural stability or transposable element control. DNA template stability was assessed in duckweed using Random Amplified Polymorphic DNA (RAPD) analysis, a gel-based method in which the disappearance or appearance of amplification products relative to unexposed controls indicates DNA strand breaks, adduct formation, or structural lesions at amplification primer sites. Ozyigit et al. (2021) [68] applied this approach to *L. minor* exposed to binary mixtures of cadmium and nickel across a gradient of concentrations, demonstrating that genomic template stability values remained significantly higher than those of more sensitive aquatic comparators and declined only marginally at the highest tested concentrations (Cd: 400 μM, Ni: 400 μM). This finding indicates that *L. minor* can maintain genetic stability despite substantial metal exposure, a characteristic that likely contributes to its exceptional tolerance and hyperaccumulation capacity. It was also reported that calcium supplementation alleviated cadmium-induced toxicity, suggesting that mineral nutrient interactions may enhance cellular protection mechanisms. Whether *L. minor* additionally activates DNA repair pathways, such as base excision repair, nucleotide excision repair, or double-strand break repair via non-homologous end joining, under contaminant stress remains to be directly investigated and represents an important mechanistic gap. Although the acute mechanisms are well mapped, the chronic metabolic consequences and energetic costs associated with maintaining constitutive, high-intensity redox homeostasis are not well understood. There is a notable lack of research on whether chronic exposure to low-level pollutants leads to metabolic exhaustion or permanent shifts in the plant’s primary carbon and starch metabolism over extended treatment cycles, which could ultimately compromise biomass quality for downstream applications.

### 4.3. Organic Pollutant Metabolism

Unlike elemental metals, organic pollutants such as pharmaceuticals, pesticides, and dyes undergo complex biotransformation through a three-phase model: enzymatic transformation (Phase I), conjugation to polar molecules like sugars or amino acids (Phase II), and final sequestration (Phase III) [79,81,123]. Compounds such as metformin [23], sulfamethoxazole [82], diclofenac [73], atrazine [87], and textile dyes [102,103,104,105] are taken up efficiently and subjected to partial biotransformation via conjugation, sequestration, or microbial-assisted degradation [23,78,124,125] (Figure 2). The antibiotic ofloxacin undergoes phytodegradation in *S. polyrhiza* [79]; amoxicillin is degraded with dose-dependent phytotoxicity [80]; fluoroquinolones are biotransformed through fully characterized Phase I–III pathways in *L. minor* [81]; sulfamethoxazole is removed through duckweed-enhanced Fenton reactions across four duckweed species [82]; and ibuprofen undergoes Phase II conjugation in *L. gibba* [90]. Sucralose is uniquely metabolized by *L. minor* as a carbon source [92]. Sulfadimethoxine shows reduced toxicity following photodegradation in *L. minor* [83], and lactofen undergoes stereospecific metabolic processing [89]. The mycotoxin deoxynivalenol is enzymatically biotransformed, though its 3-keto-DON metabolite retains partial in planta toxicological potential [91]. Benzophenone-3 removal is enhanced by integration with biochar adsorption [95]. While duckweed can absorb a wide array of contaminants, including benzotriazoles [97,98] and surfactants, full mineralization is rarely achieved by the plant alone [97]. Consequently, much of the degradation efficiency is attributed to the rhizosphere microbiota. Duckweed root exudates recruit bacteria such as *Pseudomonas monteilii* or *Streptomyces* spp. [75,85], and antimicrobial-degrading rhizosphere bacteria [78] that possess metabolic machinery to mineralize recalcitrant toxins, including neonicotinoids [85], microcystins [106], phenols [100], and benzotriazoles [98]. A critical uncertainty lies in the fate and toxicity of pollutant metabolites. The parent compounds may be removed from solution, but their transformation products, either sequestered within plant tissues or released into the rhizosphere, are often not fully identified. It remains unknown if these by-products possess higher toxicity, greater environmental persistence, or different bioaccumulation profiles than the original pollutants. This leads to a central dilemma: are we observing effective detoxification, or just a shift in the chemical state and environmental compartment of the contaminant? Many compounds are converted into bound or conjugated metabolites rather than fully mineralized, raising uncertainty about their long-term environmental fate [81,109]. Without integrated metabolomic and ecotoxicological assessments, it remains unclear whether duckweed-based systems eliminate chemical risk or mask it within plant biomass and sediments.

### 4.4. Adaptation and Transgenerational Plasticity

Beyond immediate physiological shifts, duckweed exhibits remarkable transgenerational plasticity. Populations exposed to toxins over multiple clonal generations have been found to develop inherited tolerance to stressors like *Microcystis* exudates, allowing subsequent generations to maintain higher growth rates and physiological health than their predecessors [117]. Similar evidence has been reported in *S. polyrhiza*, where exposure to copper stress across multiple clonal generations generated heritable and repeatable variation in transgenerational fitness responses among genotypes. Notably, offspring derived from copper-stressed ancestors often displayed enhanced resistance to recurring copper exposure, potentially through improved protection of photosystem II, although this adaptive benefit was frequently accompanied by reduced fitness under non-stress conditions. These findings suggest that stress memory in duckweeds may involve adaptive trade-offs between resistance and growth, depending on environmental context [114]. These observations indicate that duckweed remediation systems may become more efficient over time as the population adapts to a specific wastewater profile. Nevertheless, the epigenetic and molecular drivers of this transgenerational memory remain largely unknown. Mechanistically, long-term stress memory is further supported by epigenetic evidence in *L. minor*, where environmentally induced DNA methylation changes, particularly in CG and CHG cytosine contexts, have been shown to persist for 3–12 clonal generations even after the removal of the initial stress. These heritable methylation signatures, frequently enriched in transposable elements, indicate that asexual reproduction may facilitate stable retention of epigenetic information across generations, thereby linking environmental exposure history to sustained regulation of gene expression and stress-responsive phenotypes [118]. Furthermore, it is unclear if adapting to one specific toxin provides cross-tolerance to others, a factor that is vital for designing stable, long-term bioremediation systems for dynamic industrial environments.

## 5. Duckweed-Microbiome Interactions in Phytoremediation

### 5.1. Microbial Contributions to Contaminant Transformation

Duckweed-based remediation systems are typically framed as plant-driven processes, yet accumulating evidence suggests that associated microbial communities often perform the most demanding steps of contaminant transformation (Figure 1; Table 3). Duckweed roots and fronds host diverse microbial assemblages capable of degrading organic pollutants, altering metal speciation, and promoting plant growth under stress. The floating habit of duckweeds creates a distinctive rhizosphere architecture simultaneously exposed to water, air, and light. This distinguishes duckweed systems from sediment-rooted macrophytes and may underlie their disproportionate remediation efficiency relative to biomass [15,126,127]. Culture-independent microbiome studies further demonstrate that duckweed hosts a taxonomically structured and reproducible bacterial community, with *Proteobacteria* commonly dominating across environments. Despite variation in environmental inocula, duckweed selectively assembles a stable “core” microbiome and maintains consistent community composition across species and locations, indicating strong host filtering effects. Comparative analyses further reveal that duckweed-associated bacterial communities share conserved taxonomic features with terrestrial leaf microbiomes (e.g., Arabidopsis and rice), suggesting that similar ecological principles govern leaf-associated microbial assembly across aquatic and terrestrial plants [128].

Recent mechanistic evidence further highlights that plant–microbe interactions can strongly modulate contaminant fate and physiological stress. In *L. minor*-metformin systems, both axenic and non-axenic conditions achieve similarly high removal efficiencies (>99%), indicating that the plant alone is capable of pharmaceutical transformation. However, in the absence of microbiota, plants accumulate higher levels of metformin and its metabolite guanylurea and exhibit increased oxidative stress and detoxification enzyme activity, reflecting a higher physiological burden. In contrast, microbial presence reduces internal contaminant accumulation and helps maintain plant redox homeostasis, demonstrating that microbiota play a key role in redistributing contaminant processing and alleviating phytotoxic stress [129]. Duckweed-associated bacteria possess enzymatic capacities for degrading cyanotoxins, phenols, pesticides, antibiotics, and hydrocarbons, functions that duckweeds alone execute inefficiently or only partially. For example, microbiota associated with *S. polyrhiza* actively biodegrade microcystins, indicating that cyanotoxin removal in duckweed systems is largely microbiome-mediated [106]. Similarly, phenol-resistant bacteria isolated from the rhizosphere of *L. minor* sustain high degradation rates even under phenol concentrations that suppress plant growth, suggesting that microbial tolerance thresholds often define remediation limits [100]. Engineered plant-microbe systems further emphasize microbial significance. Co-cultivation of *L. aequinoctialis* with *Pseudomonas monteilii* significantly accelerates neonicotinoid degradation by coupling plant uptake with bacterial catabolic pathways [85]. Comparable functional coupling is observed in nitrogen-rich systems, where heterotrophic nitrifying bacteria and *L. gibba* mutually enhance growth and ammonium removal [130]. These findings correspond to earlier observations that mixed duckweed species cultures can outperform monocultures for mercury bioaccumulation, implying that functional complementarity, whether plant-plant or plant-microbe, can exceed single-organism performance [56]. A persistent gap in the field is the quantitative separation of plant and microbial contributions to remediation, leaving unclear whether duckweed acts as a primary detoxifier or an ecological facilitator of microbial metabolism.

**Table 3:** Duckweed-microbiome interactions with demonstrated phytoremediation relevance.

Duckweed Species	Microbial Partner	Contaminant/ Function	Outcome	References
*L. aequinoctialis*	*Pseudomonas monteilii* FC02	Neonicotinoids (dinotefuran, thiacloprid, imidaclothiz)	Accelerated degradation by coupling plant uptake with bacterial catabolic pathways; >90% removal achieved	[85]
*S. polyrhiza*	Mixed rhizosphere microbiota	Microcystins (cyanotoxins)	Microbiome-mediated biodegradation; plant alone is insufficient for cyanotoxin removal	[106]
*L. minor*	6 phenol-resistant bacterial strains (rhizosphere)	Phenol	High degradation maintained at concentrations that suppress duckweed growth; microbial tolerance defines remediation limit	[100]
*L. gibba*	Heterotrophic nitrifying bacteria	Ammonium (aquaculture water)	Mutual growth promotion; enhanced ammonium removal via plant-bacteria synergy	[130]
*L. minor*	*Paenibacillus illinoisensis* Y11 (endophyte)	Cadmium	Enhanced plant growth and Cd remediation; endophytic colonization confirmed under controlled conditions	[52]
*L. minor*	Microbiota (axenic vs. non-axenic)	Metformin + guanylurea metabolite	Both conditions >99% removal; microbiota reduce internal contaminant accumulation, oxidative stress, and detox enzyme activity	[23,129]
*L. minor*	*Streptomyces* spp. consortium (mining environments)	Heavy metals (multiple)	Microbial consortium from mining sites enhances phytoremediation potential of *L. minor*	[75]
*Multiple* *duckweed*	Mixed microbial community (diversity effect)	Heavy metals (multiple)	Duckweed species diversity decreases heavy metal toxicity by reshaping microbial metabolic functions	[43]
*L. minor*	Rhizosphere microbiome	Benzotriazole	Plant-microbiome partnership mediates benzotriazole transformation across freshwater urbanization gradient	[98]
*L. aequinoctialis*, *S. polyrhiza*	Native bacterial consortium	Chromium (batik industry effluent)	Bacterial consortium boosts duckweed biomass and enhances chromium reduction from industrial effluent	[69]
*L. minor*	Microbiota (absent, axenic condition)	Metformin + guanylurea	Without microbiota: higher internal metformin and guanylurea accumulation, elevated detox enzyme activity, increased oxidative burden, highlighting microbial role in redistributing processing	[129]
*L. minor*	Antimicrobial-degrading rhizosphere bacteria	Antimicrobials (multiple)	Photolysis and microbial breakdown act in tandem; fate of antimicrobials in wastewater treatment systems characterized	[78]

### 5.2. Duckweeds as Ecological Scaffolds for Recruitment and Functional Modulation of Microbiomes

As in many plant species, duckweeds influence microbiome assembly through root exudation, oxygen release, and nutrient fluxes that create selective microhabitats for microbial colonization (Figure 2). However, the floating aquatic lifestyle of duckweeds generates a unique interface simultaneously exposed to water, air, and light, potentially producing microbial interaction networks distinct from those of sediment-rooted or terrestrial plants. Within these microenvironments, duckweeds can enrich microbial taxa associated with nitrification, metal chelation, xenobiotic degradation, and stress mitigation. Pollutant exposure alters the composition of duckweed exudates, reorganizing microbial community structure and enabling the recruitment of microbes with specific functional capacities, including antibiotic degradation and metal sequestration [15,126,131,132]. Experimental evidence shows that duckweed diversity itself can reduce heavy metal toxicity by reshaping microbial metabolic functions rather than by increasing plant uptake alone [43]. This observation parallels the well-documented, species-specific variation in remediation performance, where distinct duckweed taxa achieve different functional outcomes under identical exposure conditions [56,109]. However, the mechanisms underlying microbiome recruitment remain unclear. Are changes in microbiome composition actively regulated through plant signaling and adaptive exudation, or are they passive consequences of stress-induced metabolic disruption? Time-series analyses of duckweed cultivation systems reveal strong feedback between plant growth and microbial community structure, with causality often difficult to unravel [133]. Moreover, microbiome composition can shift substantially across environmental gradients, challenging the reproducibility of laboratory-optimized systems under field conditions [134,135]. Without mechanistic insight into recruitment and stabilization, microbiome-driven remediation remains difficult to predict or control.

## 6. Challenge of Translating Lab Findings to Field Applications

### 6.1. Mixture Toxicity and the Limits of Single-Stressor Assays

Most mechanistic insights into duckweed-based remediation originate from controlled, single-pollutant experiments designed to isolate uptake or detoxification pathways. However, real aquatic environments expose duckweeds to complex and dynamic pollutant mixtures in which metals interact with nutrients, pharmaceuticals co-occur with pesticides, and physicochemical stressors such as light, temperature, and redox conditions fluctuate simultaneously [18,19]. Increasing evidence indicates that the effects of such combined stressors are rarely additive. Chronic low-dose exposure can impose greater cumulative physiological costs than short-term acute stress, challenging the assumption that tolerance thresholds derived from laboratory assays translate directly to field performance [26,136]. This discrepancy raises a fundamental question: how predictive are current laboratory-based assays of real-world remediation outcomes? It has been shown that synergistic toxicity can overwhelm antioxidant and detoxification pathways even when individual contaminants appear mild in isolation [76]. Conversely, some pollutant combinations unexpectedly reduce toxicity, revealing emergent system-level properties that cannot be inferred from single-stressor studies alone [73]. These findings integrate to show that duckweed performance is a function of contaminant properties, the complexity of its environment (interaction structure), temporal exposure patterns, and inherent growth-defense trade-offs. As a result, single-stressor assays may systematically overestimate remediation capacity under environmentally realistic conditions.

### 6.2. Climate Modifiers, System Design, and System-Level Trade-Offs

Environmental modifiers further complicate mixture-driven responses. Elevated temperatures may accelerate contaminant uptake while simultaneously intensifying oxidative stress, whereas elevated CO_2_ can stimulate biomass accumulation yet dilute detoxification capacity on a per-unit-biomass basis [73] (Figure 1). Seasonal variability affects duckweed physiology, as well as microbial activity, nutrient cycling, and contaminant speciation, resulting in pronounced temporal fluctuations in remediation efficiency even within the same site [26,137]. These dynamics suggest that remediation performance is shaped as much by environmental context as by species identity or intrinsic uptake capacity. System design introduces an additional and often unrecognized layer of control. Engineered configurations, including duckweed ponds, baffled reactors, constructed wetlands, and hybrid adsorption-phytoremediation systems, strongly influence hydrodynamics, oxygen availability, residence time, and microbiome structure [59,138]. In several cases, system architecture exerts a stronger influence on contaminant removal than duckweed species choice alone, indicating that ecological and engineering constraints may dominate biological potential. At the same time, trade-offs between productivity, stress tolerance, and secondary outcomes such as greenhouse gas emissions highlight the limitations of single-metric optimization strategies. Collectively, these observations depict a central gap in duckweed remediation research: the absence of predictive frameworks that integrate mixture toxicity, climate variability, microbial dynamics, and system design. Without models capable of capturing these interactions and explicitly addressing growth-detoxification trade-offs, duckweed-based remediation is likely to remain highly context-dependent, limiting its scalability and reliability under changing environmental conditions.

## 7. From Phytoremediation to Circular Bioeconomy Platforms

### 7.1. Duckweed Remediation Systems as Bioeconomy Gateways

Duckweed-based remediation has traditionally been evaluated through the narrow lens of pollutant removal efficiency. However, the rapid growth, high nutrient content, and ease of harvesting that make duckweeds effective phytoremediators also position them as biomass platforms capable of linking water treatment to downstream resource recovery. Early system-level analyses emphasized duckweed productivity and suitability for integration into engineered wastewater treatment systems, predicting their potential role in sustainable bioeconomy frameworks [139]. More recently, integrated duckweed microbial biorefinery concepts have demonstrated that remediation and biomass upscaling can be co-designed, particularly in nutrient-rich waste streams where contaminant burdens are moderate [135] (Figure 1). The feasibility of such circular strategies depends critically on contaminant composition in agro-industrial and wastewater-derived cultivation systems and the concentration-dependent tolerance thresholds of the duckweed species employed. Markou et al. (2018) [140] highlighted that agro-industrial waste streams can serve as nutrient-rich substrates for duckweed and microalgal cultivation, supporting high biomass productivity; however, these feedstocks often contain contaminants such as heavy metals, pathogens, and xenobiotics, including antibiotics and hormones. These contaminants can accumulate in harvested biomass, raising significant concerns regarding its safety for feed, food, or other reutilization pathways. Although pretreatment approaches such as anaerobic digestion and post-treatment processes (e.g., solid–liquid separation and dilution) can substantially reduce microbial and organic contaminant loads, the persistence of certain xenobiotics remains uncertain [140]. This raises an unresolved question: can biomass production and contaminant sequestration be co-optimized, or are these inherently competing objectives? Biomass reutilization pathways illustrated in Figure 1 should therefore be understood as conditional target outcomes under appropriately managed, low-contamination cultivation conditions, not as universal endpoints applicable across all remediation contexts. Duckweed tissues that accumulate nutrients may be suitable for downstream processing, yet the same physiological traits that enable efficient nutrient uptake also facilitate the accumulation of metals and other contaminants. Most current studies emphasize either remediation efficiency or biomass utilization, but rarely quantify trade-offs between these goals across different contamination contexts. These studies suggest that duckweed systems need not terminate at pollutant removal, but can serve as entry points into circular nutrient, carbon, and energy loops. This conceptual shift, therefore, raises a fundamental design problem: should duckweed systems be optimized as remediation tools that produce biomass incidentally, or as biomass-production platforms where remediation duties constitute a primary risk to be managed?

Pilot-scale evidence for integrated duckweed bioeconomy systems is beginning to emerge, though it remains limited in scope. Guo et al. (2025) [31] reported a pilot-scale duckweed-CO_2_ bioremediation platform that achieved simultaneous nutrient removal (28–70% total nitrogen and 120–148% total phosphorus), carbon capture through CO_2_ supplementation, and sustained biomass productivity of around 12 g dry weight m^−2^ day^−1^ from real domestic sewage and agricultural wastewater, demonstrating that multi-function circular outputs are achievable under non-laboratory conditions. Similarly, Hemalatha and Mohan (2022) [135] demonstrated duckweed biorefinery integration with dairy wastewater treatment at bench-to-pilot scale, coupling remediation with microbial protein production. However, it is critical to acknowledge that industrial-scale deployment of duckweed circular bioeconomy systems has not yet been demonstrated. Most published evidence remains at laboratory or small pilot scale (<1 m^2^ cultivation area), and the transition to industrial scale introduces engineering challenges, including consistent hydraulic loading, contamination event management, harvesting automation, and regulatory compliance that have not been systematically addressed in the duckweed literature. Another critical and frequently under-reported parameter in circular system design is the concentration-dependent tolerance threshold of the duckweed species employed. Growth inhibition in *L. punctata* has been reported at cadmium concentrations as low as 1–5 mg/L in short-term assays, rising to 10–20 mg/L in genotypes screened for enhanced tolerance [45,68]. A fundamental operational constraint of duckweed-based remediation systems could be treating high-load industrial effluents, which requires dilution, staged exposure, or pretreatment (e.g., chemical precipitation to reduce metals below phytotoxic thresholds) before duckweed cultivation can be productive. Van Dyck et al. (2021) [26] further demonstrated that *L. minor* growth is strongly influenced by environmental variables, including temperature, light intensity, photoperiod, and nutrient availability, emphasizing that remediation performance is highly context dependent. Their experimentally validated growth model suggests that estimates derived from standardized laboratory assays may not fully reflect biomass production and remediation potential under field conditions. These constraints must be explicitly incorporated into any circular bioeconomy design framework for duckweed remediation.

### 7.2. Contaminant Carryover and System Coupling

The same physiological traits that enable duckweeds to accumulate nutrients efficiently also facilitate uptake of metals and other persistent contaminants. This creates a puzzle at the core of duckweed-based circular strategies, as maximizing remediation efficiency may actively undermine safe biomass reuse. Integrating duckweed remediation with downstream bioenergy or bioelectrochemical platforms has been proposed to buffer economic costs while enhancing treatment efficiency. For example, duckweed has been successfully incorporated into macrophyte-integrated sediment microbial fuel cells, where plant-microbe interactions simultaneously enhanced metal removal and electricity generation [42]. Similarly, duckweed-based biorefineries have demonstrated potential for coupling wastewater remediation with microbial protein production, highlighting system-level synergies between plant uptake and microbial metabolism [135]. Integrated systems such as duckweed-coupled microbial fuel cells highlight this tension by simultaneously enhancing metal removal and biomass productivity, while leaving open questions about the fate of accumulated contaminants in residual biomass and byproducts [42]. While coupling wastewater remediation with microbial protein production or energy recovery can improve system economics, these pathways implicitly assume that contaminant carryover is either negligible or manageable [135]. In practice, however, few studies trace contaminants across harvesting, processing, and post-processing stages. Without this accounting, complexity risks becoming conceptual rather than operational. This indicates a key sustainability gap: bioeconomy integration requires not only biomass yield, but contaminant fate transparency. Two major barriers hinder the safe circular use of duckweed biomass: a regulatory void due to undefined safety thresholds for reuse and an unresolved technical question about contaminant mobilization during downstream processing [140,141]. Metals and other persistent compounds may partition into residual biomass, digestate, or solid byproducts, potentially creating secondary waste streams rather than closed nutrient loops. At present, few studies explicitly track contaminant fate across harvesting, processing, and reuse stages, limiting confidence in claims of sustainability. This gap is especially problematic for large-scale deployment, where regulatory scrutiny and environmental risk assessment become unavoidable.

A critical and underexplored dimension of circular bioeconomy integration is the speciation and residual bioaccessibility of contaminants in harvested duckweed biomass. Heavy metals accumulated in duckweed tissues exist in multiple chemical forms: soluble ionic fractions in the cytoplasm, cell wall fractions (typically as pectate complexes), vacuolar-sequestered forms (often as metal-phytochelatin complexes), and extracellular plaques on root surfaces, each with fundamentally different bioaccessibility profiles. Wang et al. (2021) [53] demonstrated through sequential extraction analysis of cadmium-exposed *L. punctata* that 21–54% of accumulated Cd resided in cell wall-bound fractions, with 14–23% in cell organelle pools. The bioaccessibility of these fractions to gastrointestinal digestion (if biomass enters a feed or food chain) or to leaching during biogas digestate processing differs substantially between chemical forms. Metals bound in cell wall fractions may be released and become bioavailable during enzymatic hydrolysis, thermal processing, or anaerobic digestion, potentially increasing bioaccessibility in processed biomass relative to raw plant material in ways that are not captured by total elemental analysis of harvested fronds. This dynamic is rarely characterized in duckweed biorefinery studies, leaving the actual toxicological risk of processed biomass poorly defined. Furthermore, organic contaminants such as pharmaceuticals and their Phase II conjugates may be released during composting or anaerobic digestion, representing a risk that is distinct from the risks associated with heavy metals and cannot be assessed by the same analytical frameworks [140,141].

Life-cycle assessment (LCA) is an essential but largely absent analytical tool in duckweed bioeconomy evaluation. Without LCA, it is not possible to determine whether the energy inputs for pumping, mixing, heating, and harvesting, the water inputs for biomass washing and processing, and the chemical inputs for pretreatment and sterilization are offset by the environmental benefits of contaminant removal and resource recovery. The few available LCA-adjacent analyses of aquatic macrophyte-based treatment systems suggest that energy balance and nutrient recovery efficiency are highly sensitive to system scale, biomass composition, and downstream processing route [141], but comprehensive LCA frameworks specifically addressing contaminated duckweed biomass valorization, integrating contaminant fate, processing losses, secondary waste generation, and end-of-life management, do not yet exist in the published literature. Ujong et al. (2025) [141] provide a farm-to-fork assessment of duckweed’s food and biorefinery potential that partially addresses this gap but focuses primarily on clean cultivation conditions rather than the contaminated biomass scenarios central to phytoremediation applications. The absence of LCA frameworks specifically designed for phytoremediation-derived biomass represents a critical knowledge gap that must be addressed before any regulatory submission or commercial development of duckweed circular bioeconomy systems can proceed on an evidence-based basis.

From a regulatory standpoint, no internationally harmonized safety thresholds currently exist for heavy metal or xenobiotic content in duckweed-derived animal feed, fertilizer, or food products. In the European Union, maximum residue limits for heavy metals in animal feed are established for common feedstuffs, but no feed-specific limits have been defined for aquatic macrophytes or duckweed-derived protein concentrates [142]. In the United States, the FDA has not established specific tolerance levels for duckweed-based food ingredients containing environmental contaminants, and GRAS (Generally Recognized as Safe) determinations for duckweed food applications have only recently been pursued for clean-cultivated product streams. In the absence of species- and contaminant-specific regulatory thresholds, claims of safe circular bioeconomy applications for phytoremediation-derived duckweed biomass remain formally unverifiable [140,141,142]. This regulatory void is particularly acute for pharmaceutical contaminants, for which no feed or food residue limits have been established for plant-based matrices. Field-scale deployment of duckweed circular systems will therefore require not only technical demonstration, but proactive regulatory engagement and the development of novel safety frameworks tailored to phytoremediation-derived biomass, a process that will require significant coordination between academic, industrial, and regulatory stakeholders.

### 7.3. Design for Separation to Develop Adaptive Circular Systems

Moving duckweed remediation toward true bioeconomy integration will require a shift from efficiency-centric design to separation-aware system architecture. The transition from phytoremediation to circular bioeconomy platforms ultimately depends on system design. Engineered configurations that prioritize biomass recovery may require different operational strategies than those optimized for maximal contaminant removal, reflecting earlier observations that system architecture can outweigh species identity in determining remediation outcomes [139]. This raises a strategic design question: should duckweed systems be operated as remediation-first platforms with biomass as a secondary output, or as production systems constrained by contamination risk? Earlier sections of this review demonstrate that remediation performance is shaped as much by system configuration as by species identity. The same principle applies to biomass upscaling. Systems may need to spatially or temporally partition duckweed populations, allocating some cohorts to high-risk contaminant sequestration and others to low-risk biomass recovery. Such design logically aligns with broader trends in engineered ecological systems, where adaptive operation replaces static optimization [139]. Hybrid platforms that integrate duckweeds with microbial metabolism already hint at this possibility but remain largely evaluated as proof-of-concept demonstrations rather than as scalable, regulated infrastructures [42,135]. A central unresolved question, therefore, remains: can duckweed systems be deliberately engineered to switch between remediation-dominant and bioeconomy-dominant modes without losing ecological robustness? Addressing this will require life-cycle-oriented experimentation, explicit contaminant tracking, and closer alignment between plant biology, systems engineering, and sustainability assessment. Despite growing enthusiasm for circular integration, comprehensive life-cycle assessments remain rare for duckweed-based systems. The absence of quantitative frameworks linking contaminant uptake, biomass utilization, and environmental risk constrains rational decision-making. Without such analyses, duckweed remediation risks rolling between two extremes: overly conservative disposal of potentially valuable biomass, or premature promotion that underestimates contaminant persistence.

## 8. Conclusions

**Figure 3:**
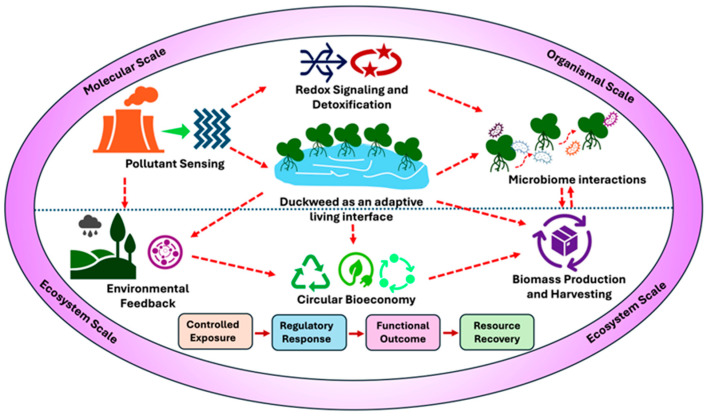
Multi-scale framework illustrating duckweed-mediated phytoremediation as an adaptive biological interface linking molecular, organismal, and ecosystem-level processes. Pollutant sensing initiates redox signaling and detoxification pathways that coordinate physiological responses, microbiome interactions, and biomass production. Environmental feedback and ecological interactions influence remediation outcomes and circular bioeconomy applications. The framework highlights the progression from controlled contaminant exposure to regulatory responses, functional remediation outcomes, and resource recovery, emphasizing the integration of molecular regulation, ecological dynamics, and sustainable biomass utilization. Solid arrows (red and green) indicate direct, linear process flow, while dashed arrows (red) represent bidirectional or multidirectional interactions and indirect relationships among the biological and ecological components.

Duckweeds offer a simple but powerful example for environmental biotechnology that small plants can support complex and adaptable ecological functions. Despite their morphological simplicity, they integrate pollutant perception, redox signaling, metabolic detoxification, and microbiome-mediated transformation across molecular, organismal, and ecosystem scales. An experimental framework linking pollutant exposure, regulatory responses, functional outcomes, and resource recovery across molecular, organismal, and ecosystem scales is illustrated in Figure 3.

Rather than functioning as passive sinks that accumulate contaminants until saturation, duckweeds could coordinate molecular and ecological responses to pollutant exposure and to chemically variable environments. This perspective infers critical knowledge gaps. How are chemically diverse pollutants perceived and distinguished at the molecular level? How are detoxification pathways prioritized under simultaneous and often competing stresses? And how do plant-microbe consortia assemble, stabilize, and persist in open, fluctuating environments typical of real-world remediation systems? A key unresolved challenge concerns the stability and persistence of beneficial plant-microbe associations. While inoculation with growth-promoting or metal-tolerant bacteria such as endophytic *Paenibacillus illinoisensis* can significantly enhance duckweed growth and cadmium remediation under controlled conditions, it remains uncertain whether these associations persist in open systems [52]. This uncertainty reflects earlier findings of strong genotype-dependent responses to pollutants in duckweeds, suggesting that host genetic background may interact with microbiome composition to profile remediation outcomes [113]. Emerging evidence of transgenerational plasticity in *L. minor* exposed to cyanobacterial exudates further complicates this phenomenon, raising the possibility that stress memory influences not only plant physiology but also microbiome assembly [117]. If host epigenetics and microbial inheritance interact, remediation performance may depend on historical exposure rather than present conditions alone. This poses a fundamental design question: can duckweed-microbiome systems be rationally engineered and stabilized under field conditions, or will community composition and function be continuously reshaped by interacting ecological processes such as environmental selection, priority effects, microbial competition, host filtering, and temporal environmental fluctuations? Integrated platforms such as microbial fuel cell-duckweed hybrids and duckweed biorefineries demonstrate promising synergies between plant productivity and microbial metabolism, although their long-term robustness remains largely untested [42,135]. A central message of this review is that duckweed-based remediation can be governed by integrated biological and ecological processes that extend beyond simple uptake metrics. Species diversity, genetic and regulatory plasticity, microbiome interactions, environmental complexity, and system design could jointly determine performance. These multilayered interactions can reveal duckweeds not as static treatment agents, but as systems whose remediation potential emerges from coordinated plant-microbe-environment feedback.

## Figures and Tables

**Table 1:** Species-dependent duckweed remediation performance across contaminant classes.

Pollutant/ Contaminant	Species	Mechanisms	References
Gold	*L. aequinoctialis*	Metal uptake in planta reduction to nanoparticle formation	[37]
Selenium	*L. punctata*	Altered antioxidant responses and metabolism	[38]
Copper	*L. minor*	Possible stimulation of transport or redox processes	[39]
Manganese, Chromium	*S. polyrhiza*	Enzymatic antioxidant modulation under metal exposure	[40]
Cobalt	*L. minor*	High enzymatic and non-enzymatic antioxidants activity	[41]
Heavy metals	*L. minor*	Removal via adsorption and electrochemical reduction steps	[42]
Heavy metals	*L. aequinoctialis*, *S. polyrhiza*	Microbe-mediated enhanced antioxidant enzyme activity	[43]
Arsenic, Iron	*L. minor*	Chelators increase Arsenic bioavailability and uptake	[29]
Cadmium	*L. punctata*	Ammonium enhanced Cadmium cell wall immobilization	[44]
Cadmium	*L. punctata*	Bioaccumulation of Cadmium	[45]
Heavy metals	*L. aequinoctialis*	Uptake and bioaccumulation of metals	[32]
Cadmium	*L. punctata*	DNA repair, ROS, and vacuolar sequestration involvement	[46]
Gadolinium	*L. gibba*	Biosorption and recovery of ionic gadolinium	[47]
Cadmium	Duckweed	GO promoted the accumulation of Cadmium in duckweed	[48]
Lead	Duckweed	High bioavailability at pH 5.0–7.0 (mildly acidic to neutral)	[33]
Lead	*L. minor*	Oxalic acid treatment caused Lead higher accumulation	[30]
Copper	*L. minor*	Bioabsorption of copper	[49]
Heavy metals	*L. aequinoctialis*, *L. gibba*	Multi-metal phytoextraction through accumulation	[50]
Chromium	*L. minor*	Humic acid significantly reduced chromium bioavailability	[51]
Cadmium	*L. minor*	Microbial synergy enhances heavy-metal remediation	[52]
Cadmium	*L. punctata*	Cell wall sequestration and vacuolar compartmentalization	[53]
Heavy metals	*L. punctata*	Antioxidant enzyme and starch metabolism changes	[54]
Heavy metals	*W. brasiliensis*, *L. minuta*	Coupling phytoremediation with biomass valorization	[55]
Mercury	*L. minor*, *S. polyrhiza*	Synergistic accumulation	[56]
Nickel	*L. minor*	Chelation and bioaccumulation	[57]
Heavy metals	*L. minor*	Metal chelation and sequestration	[58]
Boron	*L. gibba*	Uptake and compartmentalization	[59]
Arsenic	*L. valdiviana*	Adsorption and detoxification	[60]
Heavy metals	*L. minor*	Citric acid enhanced heavy metals uptake and accumulation	[61]
Heavy metals	*L. minor*	Species-specific uptake efficiency for diverse contaminants	[62]
Heavy metals	*L. minor*	Phytochelatin-mediated sequestration	[63]
Selenium	*L. minor*	Chemical speciation governs bioavailability	[64]
Mercury	*L. gibba*, *L. minor*, *S. polyrhiza*	Bioaccumulation varies among different species	[65]
Arsenic	Duckweed	Soil amendment decreased arsenic uptake in rice grain	[66]
Heavy metals	*L. gibba*	Accumulation and biosorption	[67]
Cadmium, Nickel	*L. minor*	Antioxidant defense and metal chelation mechanisms	[68]
Chromium	Duckweed	Plant-microbe synergy improves chromium reduction	[69]
Lead	*L. trisulca*	Pectinous cell wall thickenings help against lead toxicity	[70]
Heavy metals	*L. minor*	Metal-specific transport and sequestration	[71]
Zinc	*L. minor*	CO_2_-enhanced growth improves metal extraction	[72]
Cadmium	*L. minor*	Thiol-based chelation and CO_2_-driven growth stimulation	[73]
Heavy metals	*L. minor*	Biomass density governs metal uptake capacity	[74]
Heavy metals	*L. minor*	Microbial mobilization and plant uptake synergy	[75]
Heavy metals	*L. minor*	Reversible binding to cell walls	[76]
Uranium, Thorium	*L. minor*, *L. gibba*	Surface adsorption and intracellular sequestration	[77]
Antimicrobials	*L. minor*	Uptake, photolysis, and microbial breakdown	[78]
Oxytetracycline	*L. aequinoctialis*	Activation of degradation pathways	[24]
Ofloxacin	*S. polyrhiza*	Phytodegradation of antibiotic	[79]
Amoxicillin	*S. polyrhiza*	Degradation of antibiotic	[80]
Fluoroquinolones	*L. minor*	Transformation of fluoroquinolones	[81]
Tetracycline	*L. minor*	Biosorption of drugs by the duckweed	[27]
Anti-HIV drugs	*L. minor*	Increased activity of P450 and antioxidant enzymes	[28]
Antibiotics	*L. minor*	Synergistic toxicity affecting photosynthesis	[22]
Sulfamethoxazole	*L. aequinoctialis*, *L. minor*, *L. punctata*, *S. polyrhiza*	Fenton reactions enhanced by duckweed	[82]
Sulfadimethoxine	*L. minor*	Reduced toxicity after photodegradation	[83]
Terbuthylazine	*L. minor*	Antioxidant enzymes’ introduction improved removal rates	[84]
Neonicotinoid	*L. aequinoctialis*	Microbe-assisted enhanced biodegradation	[85]
Pesticides	*S. polyrhiza*	Increased light intensity reduces remediation efficiency	[86]
Herbicides	*L. minor*, *S. polyrhiza*	Herbicide uptake and detoxification	[87]
Herbicides	*S. polyrhiza*	Temperature modulates photosynthesis and detox capacity	[88]
Lactofen	*L. minor*	Stereo-specific metabolic pathways	[89]
Metformin	Duckweed	Removal via uptake and transformation	[23]
Ibuprofen	*L. gibba*	Biotransformation via conjugation	[90]
Diclofenac	*S. polyrhiza*	Tolerance enables pharmaceutical phytoremoval	[25]
Deoxynivalenol	*L. minor*	Enzymatic biotransformation of deoxynivalenol	[91]
Sucralose	*L. minor*	Uptake and metabolization of sucralose as carbon source	[92]
Galaxolide	*L. minor*	Oxidative stress and detox enzyme activation	[93]
NAP and MC-LR	*L. punctata*	Stronger antioxidant responses post exposure	[94]
Benzophenone-3	*L. minor*	Biochar adsorption enhances plant uptake	[95]
LAS	*L. minor*	Activation of LAS degradation pathway	[96]
Benzotriazoles	*L. minor*	Abiotic (photolysis) and biotic pathways act in tandem	[97]
Benzotriazole	*L. minor*	Microbiome-mediated benzotriazole transformation	[98]
1,4-dioxane	*L. gibba*	Partial conversion of 1,4-dioxane into amino acids	[99]
Phenol	*L. minor*	Bacterial degradation	[100]
Petrohydrocarbons	*L. paucicostata*	Biodegradation of petroleum hydrocarbons	[101]
Basic Green 4	*L. gibba*	Likely oxidative enzymes and sequestration	[102]
Brilliant Blue	Duckweed	Synergistic removal through adsorption and breakdown	[103]
Acid Bordeaux B	*L. minor*	Activation of biodegradation pathway	[104]
Toluidine Blue	*L. minor*	Surface binding and uptake processes	[105]
Microcystins	*S. polyrhiza*	Microbial biodegradation-mediated detox	[106]
Microplastics	*L. minor*	Surface adhesion and entrapment	[107]
ZnONP	*L. minor*	Antioxidant response for nanotoxicity mitigation	[108]
CECs	*W. arrhiza*, *L. minor*	Species-specific uptake and biodegradation	[109]
Micropollutants	*L. minor*	Exudate-mediated detox and signaling	[110]
DTB-A, DTB-M	*S. polyrhiza*	Stress-induced physiological changes	[111]

**Table 2:** Core molecular mechanisms of duckweed contaminant uptake, detoxification, and stress tolerance.

Category	Mechanism	Key Molecules/Genes	Contaminants Addressed	References
Direct phytoremediation	Transporter-mediated uptake	ZIP/IRT importers, NRAMP permeases, HMA P-ATPases	Cd, Pb, Cr, Co, Ni	[40,63]
Direct phytoremediation	Vacuolar sequestration	HMA-type pumps; tonoplast transporters	Cd, Zn, Pb	[46,53]
Direct phytoremediation	Phytochelatin synthesis	PCS enzymes; PC2–PC6 peptides	Cd, As, Pb	[58]
Direct phytoremediation	Metallothionein expression	SpMT2a and related MTs	Cd, Cu	[116]
Direct phytoremediation	Cell wall immobilization	Pectin matrix thickenings; pectate	Pb	[70]
Direct phytoremediation	Root iron plaque formation	Ferrihydrite deposits on root surface	As, Pb	[29]
Direct phytoremediation	Phase I–III organic biotransformation	CYP450s, GSTs, glucosyl transferases (green liver model)	Pharmaceuticals, pesticides, dyes	[79,81,82]
Stress tolerance	Antioxidant enzyme induction	SOD, CAT, APX, GPX, glutathione enzymes	ROS from metals/organics	[54,73]
Stress tolerance	Redox signaling & DNA protection	ROS second messengers; RAPD genomic template stability	All oxidative stressors	[68]
Stress tolerance	Epigenetic reprogramming	DNA methylation (CG/CHG contexts); heritable stress memory	Cyanobacterial exudates, Cu	[114,118]
Microbial-assisted	Rhizosphere microbial degradation	Catabolic enzymes (*Pseudomonas*, *Streptomyces*)	Neonicotinoids, microcystins, phenols, benzotriazoles	[75,85,98]

## Data Availability

The original contributions presented in this study are included in the article. Further inquiries can be directed to the corresponding author(s).
